# Fasciotomy and Debridement in Alpha-Hemolytic Associated Type II Necrotizing Fasciitis of The Hand: A Case Report

**DOI:** 10.5704/MOJ.2511.021

**Published:** 2025-11

**Authors:** V Gunawan, JM Pelealu, A Sejati, F Chriestya

**Affiliations:** 1 Department of Orthopaedic and Traumatology, Atma Jaya Hospital, Jakarta, Indonesia; 2 Department of Internal Medicine, Universitas Katolik Indonesia Atma Jaya, Jakarta, Indonesia

**Keywords:** necrotizing fasciitis, debridement, fasciotomy, alpha-haemolytic streptococcus sp

## Abstract

Necrotizing Fasciitis (NF) is a rare and potentially life-threatening infection affecting the skin and soft tissue. Its incidence is estimated at 4 cases per 100,000 people a year in the United States. Gram-positive cocci are the common causative organism. With a mortality rate of 25% to 35%, it should be diagnosed as soon as possible, and the treatment should be expedited. A 68-year-old woman presented with acute disturbed consciousness, accompanied by fever, swelling, and pain on the index finger of the right hand. She gave a history of a cut finger one day prior. Her index finger appeared pale and cold in touch with no capillary refill time and was insensate. Initially, she was diagnosed with a diabetic hand. With the disease progressivity combined with laboratory and ultrasonography results, she was diagnosed with NF, and emergency debridement and fasciotomy were done. NF is a lethal and fast progressing Skin and Soft Tissue Infections (SSTIs) that should be diagnosed early to reduce the mortality rate. A slower rate of infection does not exclude NF as a diagnosis because many factors can affect it, such as its aetiology, coexisting disease, or our empiric antibiotic. In treating NF, a blood culture should be done to identify its cause, and we should perform adequate surgical debridement with or without fasciotomy based on its manifestations.

## Introduction

Necrotizing fasciitis (NF), or flesh-eating disease, is an acute, rare, and potentially life-threatening skin and soft tissue infection (SSTI)^1^. NF is classified as type 1 (polymicrobial) or 2 (monomicrobial). It is majorly caused by gram-positive cocci (Staphylococcus aureus and Streptococci), which are introduced through a break in the skin’s integrity. Gram-negative rods such as *Klebsiella sp.* and *Escherichia coli* also serve as a NF aetiology^1,2^.

NF initially involves the deep fascia and subcutaneous fat, with the infection being spread at an alarming rate, which can cause extensive necrosis, gangrene, small vessel thrombosis, and superficial nerve destruction. Systemically, it is characterised by fever and tachycardia, and in severe infection, hypotension, mental confusion, and end-organ failure may present^1,3^. Its incidence is estimated at 0.4 – 1.3 cases per 100,000 person-year in Canada and Florida. The mortality rate by multiple reports worldwide, is 15-36% in the USA, Taiwan, and the Philippines^4^. By being female, elderly (>60 years old), or having chronic heart disease, cirrhosis, skin necrosis, tachycardia >130/min, low systolic BP (<90 mmHg), and high serum creatinine (≥1.6 mg/dL) are the mortality prognostic factor for NF^4^.

NF can be treated with an empiric regime using clindamycin and high-dose penicillin to cover gram-positive and anaerobic bacteria. Blood culture and antibiotic resistance are also needed to identify the aetiology and choose the right antibiotics. Surgical debridement is said to be the cornerstone of NF treatment. The mortality rate is nearly 100% without surgery. Even with intravenous antibiotics and surgical treatment, the mortality rate is 26.4% for those with community-acquired NF and as high as 36.3% for post-procedural necrotizing infection^1^.

## Case Report

**Fig. 1:**
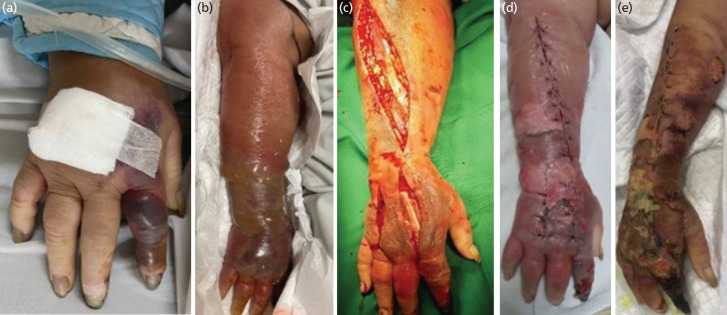
(a) Clinical appearance on admission day (two days after got injured) with the bullae only on her second finger. (b) The swelling and bullae extended to proximal forearm. (c) Fasciotomy and debridement were done on her interosseus, adductor pollicis, and extensor compartment. (d) The wound is closed loosely. (e) Slough appeared on week-2 post-operative, necrotic in second finger and no discharge coming out from incision site.

A 68-year-old woman presented with acute disturbed consciousness 2 hours before admission, accompanied by fever, swelling, and pain on the index finger of the right hand 1 day before admission. She had hypertension and type II diabetes mellitus (T2DM) with a medical history of Candesartan 8mg, Metformin 500mg, and Clopidogrel 75mg. Her index and middle fingers of her right hand got cut by a knife, and the next day, it became swollen and painful (Fig. 1a). Her index finger appeared pale and cold in touch, with no capillary refill time, and was insensate on the distal and middle phalanx.

**Fig. 2:**
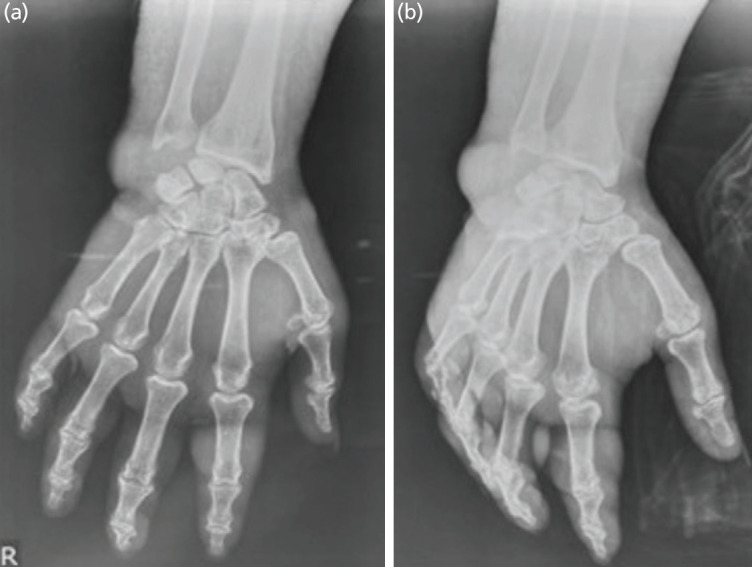
(a and b) Radiograph of the right hand showed soft tissue swelling.

She was admitted with hypotension and did not respond to fluid challenges. She was immediately given a vasopressor (norepinephrine). Laboratory results showed a leucocytosis (20,440/mm^3^), increased erythrocytes sedimentation rate (ESR) at 55mm/h, and 129 mg/dL for random blood glucose. Radiographs of the right hand showed soft tissue swelling (Fig. 2). She was diagnosed with a septic shock due to a diabetic hand.

**Fig. 3:**
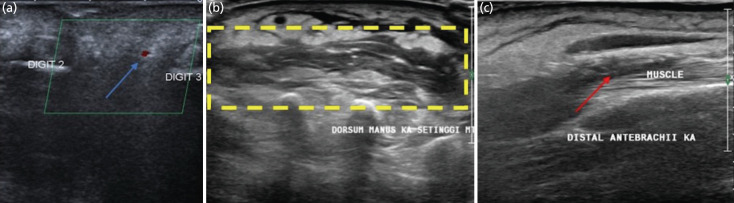
(a) Doppler ultrasonography (USG) on dorsal hand showed no blood flow in radial nor ulnar side of proper digital artery of second finger, while its visible in third finger (blue arrow). (b) Focal multiple anechoic / hypoechoic area in dorsal of the hand on metacarpals level (yellow dashed square) and (c) hypoechoic in intermuscular area in distal forearm (red arrow).

She was treated in High Care Unit (HCU) with vasopressor and empiric antibiotics (meropenem and metronidazole). On day 3, the swelling extended proximally with large bullae. The antibiotics were changed to ampicillin/sulbactam and metronidazole. The next day, her consciousness deteriorated as she only responded to pain. The swelling and bullae also extended to her proximal forearm (Fig. 1b). Eventually, she was referred to Orthopaedics and a doppler ultrasonography (USG) was performed on her right hand and forearm (Fig. 3). Initially, a Computerised Tomography Scan (CT scan) was planned, but the hand and forearm swelling made it difficult to fit in the CT Scan machine. USG showed no blood flow on the proper palmar digital artery on the ulnar or radial side (Fig. 3a). As her consciousness and forearm condition worsened, we decided to perform an emergency fasciotomy and debridement, and a diagnosis of necrotizing fasciitis was made.

Fasciotomy and debridement were performed on the dorsal interosseus and extensor compartments. The extensor compartment was relatively healthy, but there was extensive necrotic tissue - consistent with USG findings (Fig. 3b) on the dorsal interosseus compartments. As such, debridement with necrotomy was performed. No destruction was found on the muscles and tendons. The wound was loosely closed primarily, then covered with tulle dressing and gauze.

Post-operatively, her consciousness showed improvement, and her blood pressure improved. Her blood culture results on post-operative day 2 grew Streptococcus viridans, alpha-hemolytic. Based on her blood culture and her clinical conditions, metronidazole was stopped, and ampicillin/sulbactam was continued until day ten. On postoperative day 8 (day 10 of ampicillin/sulbactam), she was discharged, and her post-operative wound is treated at homecare treatment once every two to three days. On week-2 post-operatively, the wound on her forearm was relatively healthy with no discharge, while there was slough on her dorsal hand (Fig. 1e).

Six weeks post-operatively, she was admitted again for her second surgery, at which amputation of her index finger was done. Clinically, there was a clear separation between necrotic and healthy tissue on the index finger. We proceeded to amputate her index finger at the proximal third of the proximal phalanx. Post-operatively, she could perform gripping motions well. She was discharged one day after the amputation. Four weeks after amputation, her amputation wound appeared good, with no sign of infection, and she could perform her daily activities without any hindrance.

## Discussion

*Streptococcus sp.* is the most common cause of NF, specifically beta-hemolytic. However, no single report of alpha-hemolytic-associated NF has been found. *Streptococcus sp.* is divided into three groups: alpha-hemolytic (incomplete hemolysis), beta-hemolytic (clear and complete red blood cell’s lysis), and gamma-hemolytic (no hemolysis). As the characteristics of alpha-hemolytic *Streptococcus sp.*, tissue destruction severity is not as severe as beta-hemolytic Streptococcus sp. In our patient, we did not find any muscle involvement (only subcutaneous), gangrenous tissue was limited over her proximal phalanx, and necrotic tissue only over the dorsal aspect of her hand. A retrospective study by Shivalingappa *et al* showed a more severe clinical appearance and laboratory results (extensive abscess and necrotic tissue with Hb level below 10mg/dL), and mostly caused by beta-hemolytic bacteria^4^.

Clinical scores on laboratory results can be helpful as an indicator of NF risk, and known as the Laboratory Risk Indicator for Necrotizing Fasciitis (LRINEC) (Table I)^3^. In our patient, her WBC is 20,440/mm^3^, Hb at 12.3g/dL, sodium at 133.8mmol/L, creatinine at 1.93mg/dL, and glucose at 129 mg/dL. Unfortunately, we did not perform CRP-level checks on this patient. Her LRINEC score without CRP level is 6, and it falls in the category of suspicious NF.

**Table I:** Laboratory risk indicator for necrotizing fasciitis (LRINEC) score.

Variable	Score
C-Reactive Protein (mg/dL)	
<150	0
≥150	4
White Blood Count (x10^3^ mm^3^)	
<15	0
15-25	1
≥25	2
Hemoglobin (g/dL)	
>13.5	0
11 – 13.5	1
<11	2
Sodium (mmol/L)	
≥135	0
< 135	2
Creatinine (mg/dL)	
≤1.6	0
>1.6	2
Glucose (mg/dL)	
≤180	0
>180	1

Model of LRINEC Score to predict necrotizing fasciitis. The maximum score is 13, suspect of necrotizing fasciitis if the score is ≥ 6, and strongly predictive of necrotizing fasciitis if the score is ≥ 8.

Diabetes mellitus is an important risk factor and also a major determinant of mortality^1-3,5^. Her T2DM was controlled as her random glucose level of 129mg/dL and an A1c level of 5.9%. We suggest another factor that made her presentation milder. Minini *et al* presented a similar case with Enterobacter Cloacae as the causative bacteria. However, while the systemic symptoms appeared milder with a higher LRINEC score (CRP level included), the intra-operative findings were more severe than ours. The patient’s random glucose was 14.37mmol/L or 260mg/dL, which was two times higher than our patient^5^.

For surgical planning, we planned to do 2 steps of surgery:

(1) debridement and fasciotomy to control the infection at its source followed by (2) amputation. As we performed the initial surgery, we attempted to minimise the extent needed for amputation. While waiting for her 2nd surgery, we encouraged the patient and her family to do hand exercises such as hand gripping motion to improve the stiffness. By improving this, we hoped to achieve optimal post-amputation results. Six weeks after debridement and fasciotomy, a clear demarcation between healthy and necrotic tissue appeared; As such, we performed the amputation of her index finger at this level

There had been a delay in surgical treatment on initial admission due to the diagnosis of a diabetic hand and not NF, which led to a delay in referral to an orthopaedic surgeon. While NF is difficult to diagnose initially, the slower rate of infection progressivity, which was proven by her blood culture result, Streptococcus viridans, alpha-hemolytic, also contributed to the delay. Due to the delay in surgical treatment, the infection had spread proximally, and we needed to perform the debridement and fasciotomy up to her forearm. This infection would have been worse if the initial antibiotics chosen were not sensitive to beta-haemolytic *Streptococcus sp.* By doing a more comprehensive investigation, performing a more complete infection marker laboratory test, and calculating the LRINEC score, we could have diagnosed it faster and more accurately and prevented the disastrous outcome of NF.

In conclusion, NF is a lethal and fast progressing SSTIs that should be diagnosed early to reduce the mortality rate. A slower infection rate does not exclude necrotizing fasciitis as a diagnosis because many factors can affect it, such as its aetiology, coexisting disease, or empiric antibiotic. Making NF as an initial diagnosis is quite challenging; therefore, by using the LRINEC score, it can help to diagnose it faster and more accurately. In treating NF, a blood culture should be done to identify its cause and we should perform immediate adequate surgical debridement with or without fasciotomy based on its manifestations.
